# The Cell-Permeable Derivative of the Immunoregulatory Metabolite Itaconate, 4-Octyl Itaconate, Is Anti-Fibrotic in Systemic Sclerosis

**DOI:** 10.3390/cells10082053

**Published:** 2021-08-10

**Authors:** John Henderson, Sharadha Dayalan Naidu, Albena T. Dinkova-Kostova, Stefan Przyborski, Richard Stratton, Steven O′Reilly

**Affiliations:** 1 Applied Sciences, NorthuMbria University, Ellison Place, Newcastle Upon Tyne NE7 7XA, UK; john.henderson@northumbria.ac.uk; 2 Cellular Medicine, School of Medicine, University of Dundee, Dundee DD1 4HN, UK; s.z.dayalannaidu@dundee.ac.uk (S.D.N.); A.DinkovaKostova@dundee.ac.uk (A.T.D.-K.); 3 Biosciences, Durham University, South Road, Durham DH1 3LE, UK; stefan.przyborski@durham.ac.uk; 4 Institute of Inflammation, University College London, Pond Street, London WC1E 6BT, UK; r.stratton@ucl.ac.uk

**Keywords:** systemic sclerosis, metabolism, fibrosis, itaconate, Nrf2

## Abstract

Systemic sclerosis (SSc) is an autoimmune connective tissue disease that leads to skin fibrosis. Altered metabolism has recently been described in autoimmune diseases and SSc. Itaconate is a product of the Krebs cycle intermediate *cis*-aconitate and is an immunomodulator. This work examines the role of the cell-permeable derivative of itaconate, 4-octyl itaconate (4-OI), in SSc. SSc and healthy dermal fibroblasts were exposed to 4-OI. The levels of collagen Nrf2-target genes and pro-inflammatory cytokines interleukin 6 (IL-6) and monocyte chemotactic protein 1 (MCP-1) were determined. Levels of reactive oxygen species (ROS) as well as the gene expression of collagen and Cellular Communication Network Factor 2 (CCN2) were measured after transforming growth factor beta 1 (TGF-β1) stimulation in the presence or absence of 4-OI. Wild-type or Nrf2-knockout (Nrf2-KO) mouse embryonic fibroblasts (MEFs) were also treated with 4-OI to determine the role of Nrf2 in 4-OI-mediated effects. 4-OI reduced the levels of collagen in SSc dermal fibroblasts. Incubation with 4-OI led to activation of Nrf2 and its target genes heme oxygenase 1 (HO-1) and NAD(P)H quinone oxidoreductase 1 (NQO1). 4-OI activated antioxidant response element (ARE)-dependent gene expression, reduced inflammatory cytokine release and reduced TGF-β1-induced collagen and ROS production in dermal fibroblasts. The effects of 4-OI are dependent on Nrf2. The cell-permeable derivative of itaconate 4-OI is anti-fibrotic through upregulation of Nrf2 and could be a potential therapeutic option in an intractable disease.

## 1. Introduction

Systemic sclerosis (SSc) is an autoimmune idiopathic connective tissue disease that is characterised by vascular abnormalities, inflammation and fibrosis [1,2]. The primary site affected is the skin, but the lung can also be affected. Several studies have suggested a role of the immune system in the activation of the fibroblasts to differentiate into myofibroblasts that then secrete the excessive extracellular matrix that underpins the main pathology of the disease [1]. The activated fibroblasts secrete exuberant levels of extracellular matrix (ECM), become contractile and resistant to cell apoptosis, and this ultimately results in fibrosis [3]. 

What governs the activation of a myofibroblast is not clear, but it may be a result of failure to terminate normal physiological responses to various stimuli that are initially beneficial but, if not appropriately terminated, result in pathological scarring. Emerging data have suggested a role for immunometabolism in inflammatory diseases [4]. Numerous studies have suggested alterations in cellular metabolism can lead to inflammatory diseases, including, among others, rheumatoid arthritis [5]. Itaconate, a recently described metabolite derived from the Krebs cycle, which is generated by the enzyme aconitate decarboxylase 1 (ACOD1) (also known as IRG1), is enormously upregulated after TLR stimulation [6] in immune cells [7]. In a quiescent state, there are tiny amounts of itaconate, but upon exposure to lipopolysaccharide (LPS), this can be released in millimolar levels [8]. Much work has now suggested that itaconate has an immunomodulatory role and that mice deficient in IRG-1 have exacerbated immune disease induced by mycobacteria [9]. Furthermore, it has been found that treatment of neurons with itaconate alters the metabolic state, which suppresses zika virus replication rates [10], and a derivative of itaconate reduced lung inflammation [11]. Recently it was demonstrated that IRG1-deficient hepatocytes in a hepatocyte-specific knockout mouse had enhanced liver fibrosis in vivo [12], and we recently demonstrated that a cell-permeable derivative of itaconate, 4-octyl itaconate, reduced collagen in dermal fibroblasts from diffuse SSc patients [13]. This work aims to extend this observation further.

## 2. Materials and Methods

SSc patients with early diffuse SSc were included (*n* = 3). Early diffuse was defined as <2 years from first non-Raynaud′s symptoms and positive for anti-Scl70 autoantibodies. All patients were female and treatment naïve, and no patient had clinical evidence of interstitial lung disease on HRCT. The study has full ethical approval with the local research ethics committee (REC) with approval no REC/13/NE/0089 and followed the declaration of Helsinki guidelines.

A small punch biopsy was taken from the affected fibrotic area of skin using a punch biopsy with a local anaesthetic. SSc dermal fibroblasts were isolated and cultured in standard fibroblast culture media, which DMEM supplemented with 10% Fetal Calf Serum (FSC) and 1% penicillin/streptomycin (Gibco). Normal dermal fibroblasts were derived as previously described [13]. Cells were treated with 4-octyl Itaconate (4-OI) (100 µM; Sigma Aldrich, Poole, UK) dissolved in Dimethyl Sulfoxide (DMSO) vehicle (0.1% *v*/*v*), and collagen was measured by the Sircol assay according to manufacturer′s instructions (Biocolor, Belfast, Northern Ireland).

### 2.1. Quantitative RT-PCR 

Q-RT-PCR was performed as previously described and normalised to 18S housekeeping gene and shown as fold change compared to vehicle. Primer sequences are listed in Table 1.

### 2.2. Antioxidant Response Element (ARE) Luciferase Assay

The enzyme activity of ARE-driven luciferase as a readout of Nrf2 activity was performed using an ARE luciferase construct. One µg of plasmid was transfected in pre-confluent dermal fibroblasts using Effectene (Qiagen, Manchester UK). After 12 h, the media were replenished, and cells were treated either with vehicle DMSO (0.1%), 4-OI (100 µM) or sulforaphane (25 µM) for a further 24 h. The cells were then lysed, and the luciferase activity was assessed using a luminometer. Data are expressed as a percentage change with untreated plasmid only set to 100%. All experiments were performed in triplicate with four technical replicates.

### 2.3. ELISA

ELISA for both interleukin 6 (IL-6) and Monocyte Chemotactic Protein 1 (MCP-1) were performed using commercial ELISA kits (R&D Systems, Oxford, UK) according to the manufacturer′s instructions. Conditioned media were measured after stimulation of healthy dermal fibroblasts with the TLR4 agonist LPS (100 ng/mL; Invivogen, UK) with or without pre-treatment with 4-OI (100 µM) or vehicle. Data are from 3 independent experiments.

ROS were measured using the cell-permeable dye CM-H_2_DFDA (Thermo Fisher, Dartford, UK) that is cleaved internally, and subsequent oxidation yields a fluorescent reporter. The dermal fibroblasts were seeded into 96 well plates treated with CM-H2DFDA to yield the ROS reporter, then subsequently pre-treated with 4-OI (100 µM) and then treated or not with transforming growth factor beta 1 (TGF-β1) (10 ng/mL). Fluorescence was analysed using a fluorescence microplate reader. Data are shown as % ROS with untreated cells set to 100%. Data are derived from 4 independent experiments.

Normal healthy dermal fibroblasts were pre-treated for 3 h with 4-OI (100 µM) and then treated or not with TGF-β1 (10 ng/mL) or 4-OI alone for 48 h after which collagen was quantified by the Sircol assay as previously described. Metabolic analysis was performed using a Seahorse XF analyser; this measures glycolysis as a proxy for extracellular acidification rate (ECAR) as previously described [13].

### 2.4. Lactate Dehydrogenase Assay (LDH)

LDH was measured as a marker of cytotoxicity in dermal fibroblasts treated or not with vehicle or 4-OI using the cytotoxicity kit (Abcam, Ab69353) according to the manufacturer’s instructions. A total of 50% DMSO was run alongside as a positive control.

### 2.5. MicroRNA Measurements

After stimulation, total RNA was isolated using miRNeasy kit using manufactures instructions (Qiagen, Manchester, UK). miRNA was converted to cDNA using Taqman™ Advanced Reverse Transcription kit (Applied Biosystems, Dartford, UK). MiR29a was measured using Taqman™ specific primers miR29a assay id: 478587_mir (Applied Biosystems, Dartford, UK), and miR155 was also measured by Taqman primers assay id: HS01374569_m1.

RNU44 assay id: 001094 was also quantified, and the data were normalised to RNU44 and presented as fold change. Quadruplicates were used for each individual sample.

### 2.6. Immunoblotting

Primary wild-type (WT) or Nrf2-knockout (Nrf2-KO) mouse embryonic fibroblast (MEF) cells isolated from Skh-1 hairless mice [14] were maintained in a humidified 37 °C incubator with 5% CO_2_ and grown in high-glucose DMEM (#41966, Thermo) with 10% heat-inactivated fetal bovine serum (FBS) (Thermo) (*v*/*v*). The MEF cells were seeded at a density of 0.5 × 10^5^ cells per well of a 12-well plate at passage 3. Sixteen hours post-seeding, the cells were treated with either 0.1% acetonitrile (*v*/*v*) (Vehicle) or 100 μM 4-octyl itaconate (4-OI) for 24 h. Twenty four hours after treatment with the vehicle or 4-OI, the cells were either exposed to control buffer (TGF-β1 reconstitution buffer, 0.1% BSA in 4 mM HCl) or stimulated with 12.5 ng/mL TGF-β1 (#100-21, Peprotech) in the presence of the vehicle or 4-OI (100 μM) for 48 h. Cells were washed twice with PBS and lysed in 1 X SDS lysis buffer (50 mM Tris HCl pH 6.8, 2% SDS (*w/v*) and 10% glycerol (*v/v*)). Protein content was measured using the BCA assay (Thermo, Dartford, UK), lysates were incubated with 5% (*v*/*v*) B-mercaptoethanol for 30 min at RT. Equal amounts of protein (5–20 μg) from each lysate is loaded and subjected to gel electrophoresis (NuPAGE 4–12% BisTris/MOPS 20-well midi gel) (Thermo Fisher, Dartford, UK) and transferred onto 0.45 μM nitrocellulose membranes (Amersham) at 70 V for 2 h using the criterion blotter with plate electrodes (Biorad, UK). Subsequently, the membranes were blocked with 5% non-fat milk (*w*/*v*) dissolved in 0.1% Tween-20 (*v*/*v*) in phosphate-buffered saline (PBST) (Milk-PBST) for 1 h at room temperature (RT). Upon completion of blocking, membranes were incubated with primary antibodies raised against NQO1 (1:1000 dilution in Milk-PBST, #62262, CST) and GAPDH (1:20000 dilution in Milk-PBST, #60004-1-Ig, Proteintech, Manchester, UK) for 2 h at RT. The membrane used for collagen detection was washed for 5 min in PBST at RT and incubated in COL1A1 primary antibody (#AB765P, Millipore, Oxford, UK) diluted 1:1000 in 3% bovine serum albumin (BSA) (*w*/*v*) in PBST (BSA-PBST) and incubated overnight. After primary antibody incubation, the immunoblots were washed thrice every 5 min with PBST, after which they were incubated with their respective fluorescently labelled secondary antibodies (LI-COR) diluted in Milk-PBST at a 1:20000 dilution for 1 h. The blots were washed in PBST for 30 min before scanning them using the Odyssey CLx Imager (LI-COR).

## 3. Results

**Figure 1:**
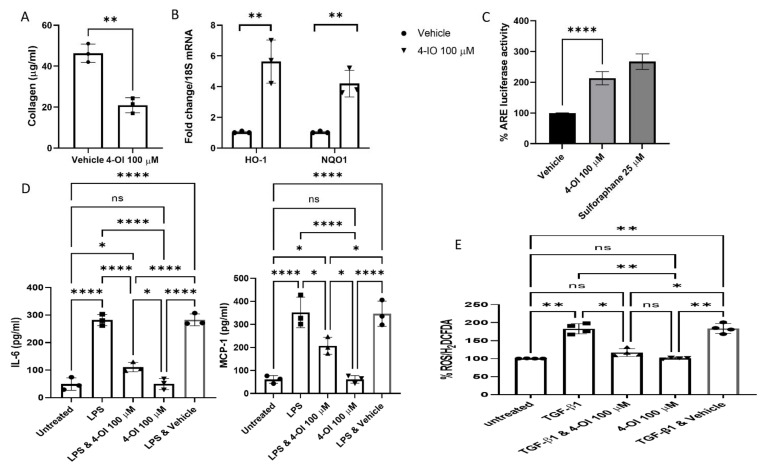
(**A**). Reduced collagen in SSc fibroblast with itaconate. SSc dermal fibroblasts were cultured in vitro with 100 µM of the cell-permeable derivative of itaconate 4-OI or not, and after 48 h, the collagen was quantified using the Sircol assay (µg/mL). Data are the mean, and error bars are the standard deviation of the mean, and each point represents a single donor. ** *p* = 0.0017 Student’s *t*-test *n* = 3. (**B**). Target genes of Nrf2 are upregulated by 4-OI. Healthy normal dermal fibroblasts were cultured with or without 4-OI (100 µM); after 24 h, the cells were harvested, and the RNA was isolated and reverse transcribed. Genes were quantified using qRT-PCR with specific primers, and data were normalised to 18S as the housekeeping gene. Data are shown as fold change to untreated cells after normalisation to the housekeeping gene 18S. Data are the mean fold change and standard deviation. Each data point represents a single sample. ** significantly difference compared to untreated cultures *p* =< 0.005 Student’s *t*-test; *n* = 3. (**C**). 4-OI activates Nrf2-dependent transcription. Dermal fibroblasts were transfected with the ARE-luciferase construct, and after 12 h, the cells were untreated or treated with 4-OI (100 µM) or the Nrf2 activator sulforaphane (25 µM). After a further 24 h, the cells were lysed, and luciferase was measured using a luminometer. Data are shown as % change with untreated (but plasmid transfected) set at 100%. Bars are the mean and standard deviation *p* = 0.0001 Student’s *t*-test *n* = 4; **** = *p* < 0.0001 Sulforaphane was excluded from this analysis as this was run only as a positive control for Nrf2 activation. (**D**). 4-OI reduces pro-inflammatory cytokine release. Heathy dermal fibroblasts were cultured and pre-treated for 3 h with 4-OI (100 µM), after which they were treated with the TLR4 agonist LPS (100 ng) for a further 24 h. Media were removed, and cytokines were measured by ELISA. Data are the mean and standard deviation IL-6 * *p* =< 0.0001 Untreated vs. LPS; * *p* = 0.0001 LPS vs. LPS & 4-OI ANOVA *n* = 3. MCP-1* *p* =< 0.0001 Untreated vs. LPS; * *p* = 0.0093 LPS vs. LPS & 4-OI ANOVA *n* = 3. (**E**). 4-OI decreased ROS levels in dermal fibroblasts. Dermal fibroblasts were cultured and preloaded with the cell-permeable dye CM-H_2_DCFDA in serum-free medium; the media were then replenished with media contained untreated, TGF-β1 only, TGF-β1 and pre-treated for 3 h with 4-OI (100 µM) or 4-OI alone (100 µM). Following a further 12 h, the cells were measured using a fluorescence plate reader. Data are expressed as % change in ROS as per H_2_DCFDA fluorescence with untreated cells set at 100%. Data are the mean and standard deviation with each point representing a single experiment. ANOVA; *n* = 4. NS = non significant.

Itaconate, whilst known to be upregulated in response to TLR stimulation, has recently been shown to be anti-inflammatory by activating Nrf2 via modification of specific cysteine residues of its principal inhibitor Kelch-like ECH-associated protein-1 (Keap1) [15]. We recently showed that the cell-permeable itaconate derivative 4-OI reduced collagen in dermal fibroblasts from diffuse SSc patients [13], and we sought to examine that further. Incubation of SSc fibroblasts with 4-OI reduced the collagen levels in the cell culture medium from these cells by ~60% compared to the vehicle-treated cells (Figure 1A) (*p* = 0.0017 Student’s *t*-test 46.3 v 20.9 µg/mL; *n* = 3).

We also found significant upregulation of the Nrf2-target genes heme oxygenase 1 (HO-1) and NAD(P)H quinone oxidoreductase 1 (NQO1) [16] in the 4-OI-treated cells (Figure 1C) (*n* = 3). It is interesting to note that Nrf2 is significantly downregulated in SSc dermal fibroblasts and skin biopsies, and Nrf2-deficient mice have exaggerated skin and lung fibrosis [17]. Nrf2 is normally associated with Keap1 through direct protein–protein interactions; this results in polyubiquitination and subsequent degradation of Nrf2 through the proteasome [18]; in this way, Keap1 constrains activation of Nrf2 and subsequent downstream gene expression. Molecules that modify Keap1 cause stabilisation and nuclear translocation of Nrf2 and subsequent cytoprotective Nrf2-dependent expression of genes containing antioxidant response elements (AREs) in their promoter sequences to restore the homeostatic balance [19]. To confirm that 4-OI induces ARE-dependent transcription, we transfected dermal fibroblasts with an ARE-driven luciferase reporter, and we could see a significant increase in ARE-luciferase activity by 2-fold (*p* = 0.0001, Student’s *t*-test, *n* = 4) (Figure 1C). The magnitude of this increase is similar to that caused by the known potent Nrf2 inducer sulforaphane (Figure 1C). 

Based on recent previous studies that have identified the role of itaconate as being anti-inflammatory in macrophages—as measured by cytokine responses after LPS stimulation [15,20]—we sought to determine the effect of 4-OI on the cytokine IL-6 and the chemokine MCP-1 after TLR stimulation. Previous studies have identified these as target proteins of TLR4 signalling in dermal fibroblasts [21]. Incubation with the TLR4 ligand LPS led to significant upregulation of both IL-6 and MCP-1 in healthy dermal fibroblasts that was attenuated with pre-treatment of cells with 4-OI (Figure 1D). This suggests that, like previous studies in immune cells [8,15], itaconate possesses anti-inflammatory properties. Both IL-6 and MCP-1 are dysregulated in SSc [22,23].

Next, we examined the levels of reactive oxygen species (ROS) after stimulation with the pro-fibrotic cytokine transforming growth factor beta 1 (TGF-β1), highly elevated in SSc [24], with prior incubation with 4-OI as Nrf2-mediated activation of the stress response would be expected to reduce this effect. Figure 1E demonstrates that pre-treatment with 4-OI reduced ROS induction by TGF-β1 significantly (*p* = 0.05 TGF-β1 versus TGF-β1 & 4-OI treatment, ANOVA, *n* = 4). 

**Figure 2:**
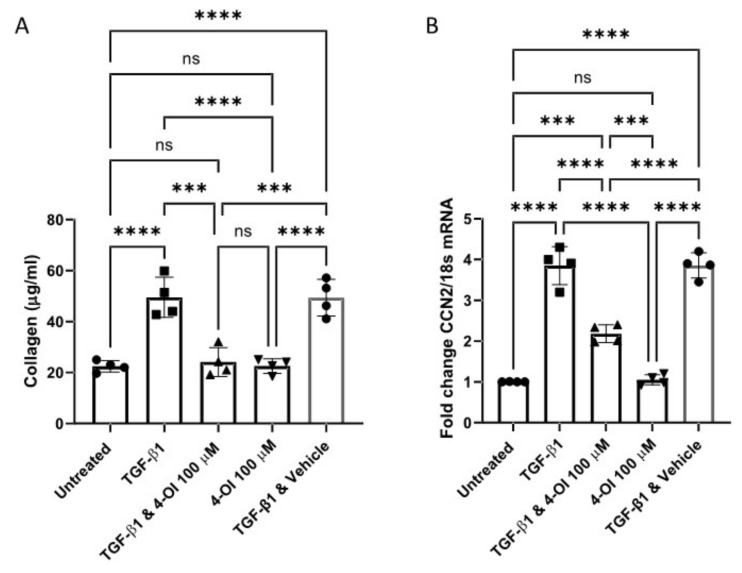
4-OI reduced TGF-β1 mediated collagen induction. (**A**). 4-OI reduces collagen induced by TGF-β1. Cells were cultured and pre-treated for 3 h with 4-OI the stimulated with TGF-β1 (10 ng/mL) or untreated and 4-OI alone and after 48 h collagen was quantified using the Sircol assay. Data are the mean and standard deviation from 3 donors. Each point is an independent experiment. * *p* =< 0.0001 ANOVA *p* = 0.0154 LPS vs. PLS & 4-OI *n* = 4. (**B**). mRNA for CCN2 was quantified by qRT-PCR after 6 h and normalised to 18S. Data are fold change compared to untreated control, which is set to 1. Data are the mean and standard deviation. Each point is an independent experiment. * *p* = 0.0001 Untreated vs. TGF-β1 treated. * *p* =< 0.0001 TGF-β1 vs. TGF-β1 & 4-OI; * *p*< 0.0001 TGF-β1 vs. 4-IO ANOVA *n* = 4; *** *p* =< 0.001, **** *p* =< 0.00001; NS = Non significant.

Additionally, pre-treatment with 4-OI significantly reduced TGF-β1 stimulation of collagen protein and CCN2 mRNA in healthy dermal fibroblasts (Figure 2A,B), suggesting that this compound has an anti-fibrotic effect given the primary role of TGF-β1 in fibrosis. This is not associated with an increase in cell death as LDH release into the media was not increased by treatment with 4-OI. Indeed, 4-OI also reduced the TGF-β1 induced glycolysis, as demonstrated by extracellular acidification. Treatment with 4-OI reduced ECAR by over 45% compared to TGF-β exposed cells.

**Figure 3:**
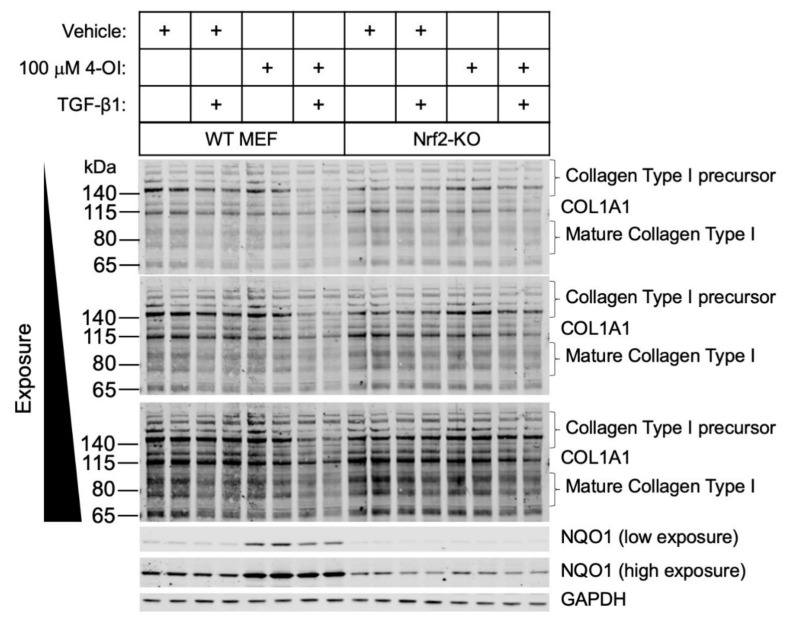
Nrf2 is required for the 4-OI-mediated decrease in collagen type I levels in primary mouse embryonic fibroblast cells. Primary WT or Nrf2-KO cells were treated with vehicle control or 4-OI and, after 24 h, stimulated with TGF-β1 (12.5 ng/mL) for a further 48 h. Cells were lysed, and cell lysates were subjected to immunoblotting with the indicated antibodies.

To determine the role of Nrf2 in mediating the effects of 4-OI in the regulation of collagen expression, we used murine embryonic fibroblasts (MEFs) from either wild-type or Nrf2-knockout mice. In wild-type cells, the collagen levels were attenuated with the addition of 4-OI (Figure 3). By contrast, the decrease in collagen by 4-OI was abolished in the Nrf2-KO cells, as was the increase in the Nrf2-target protein NQO1. This result indicates that the anti-fibrotic effect of 4-OI requires Nrf2.

**Figure 4:**
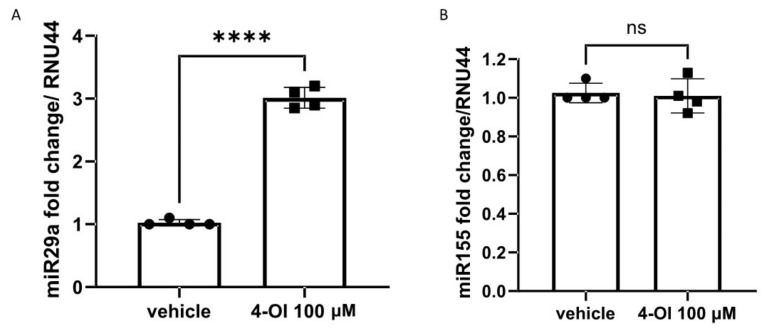
miR29a but not miR155 is upregulated by 4-OI in SSc fibroblasts. (**A**) MicroRNA29a was quantified after vehicle or 4-IO (100 µM) by Taqman PCR. Data are the mean and SD. **** *p* =< 0.001 Student’s *t*-test *n* = 4. (**B**). MicroRNA155 was quantified after vehicle or 4-OI (100 μM) by Taqman PCR. Data are the mean and SD. *n* = 4; NS = not significant.

To determine the possible mechanism of 4-OI-mediated Nrf2-dependent reduction in collagen, we sought to measure the expression of miR29a after 4-OI stimulation in SSc fibroblasts. This microRNA, miR29a, was chosen as it is a potent regulator of collagen1A1 [25,26] and a very powerful antifibrotic regulator, and also because Nrf2 has been shown to regulate this microRNA [27]. Figure 4A shows that after 4-OI incubation in dermal fibroblasts from SSc donors, there is a statistically significant ~3-fold elevation of miR29a compared to vehicle (*p* =< 0.001; Student’s *t*-test). However, treatment with 4-OI did not alter the expression of microRNA155, another important regulator of collagen [28] (Figure 4B).

## 4. Discussion

We show here that itaconate acts in both an anti-inflammatory and anti-fibrotic manner in dermal fibroblasts and that this is associated with upregulation of Nrf2-target genes and a decrease in ROS levels. The fact that Nrf2 is significantly reduced in SSc cells and skin and lung tissues [17] suggests that Nrf2 restoration through a compound such as 4-OI would be a possible therapeutic strategy. 

4-OI is a cell-permeable derivative of itaconate that has been demonstrated to alkylate cysteine residues on multiple proteins, including Keap1 [15], GAPDH [29] and (Receptor-interacting serine/threonine-protein kinase 3) RIPK3 [30]. One of the primary effects of this post-translational modification is Nrf2 activation, which in turn promotes the resolution of inflammation to restore homeostasis. Nrf2 is a central regulator of the cellular antioxidant response mediating the upregulation of cytoprotective proteins such as NQO1. Using fibroblasts from Nrf2-KO mice, we could see that the elevated collagen expression mediated by TGF-β1 was not affected by pre-treatment with 4-OI. This shows that the effect of 4-OI is at least partly dependent on Nrf2 activation, which is in accordance with previous studies in regard to inflammation [15]. Recently 4-OI has been demonstrated to reduce osteoclastogenesis in an Nrf2-dependant manner [31]. Additionally, 4-OI has recently been shown also to modify activation of the inflammasome and resultant Interleukin-1 (IL-1) release [32]. Interestingly, a very recent study has shown that mice deficient for IRG1 (the enzyme responsible for generation of Itaconate) have exacerbated fibrosis in the bleomycin mouse model of fibrosis [33]. This was attributed to the macrophage component and not the fibroblasts themselves, as reconstitution with wild-type macrophages reduced the fibrosis [33]. We also show that 4-OI reduced pro-inflammatory proteins IL-6 and MCP-1, both critical in SSc disease [22] and also the levels of ROS. ROS are high in SSc fibroblasts, whereas HO1, a target of Nrf2, is low [34]. Due to the high levels of ROS and significantly reduced levels of Nrf2 in SSc [35], the anti-fibrotic mechanisms could be impaired; indeed, Nrf2-KO mice have exacerbated fibrosis in the bleomycin model of skin fibrosis [35]. Thus, pharmacological strategies that restore diminished Nrf2, such as 4-OI, would be a useful therapeutic. 

Mechanistically, we examined the levels of microRNA29a after 4-OI treatment and found them to be elevated. MicroRNA29a was chosen as it is a critical and powerful regulator of fibrosis directly targeting collagen 1 in its 3′UTR [36] and is significantly reduced in SSc fibroblasts [36]. Interestingly, miR155 is also a key regulator of fibrosis but was not altered by 4-OI treatment, suggesting that there is a specificity in the response. This specificity may be due to the fact that miR29 is a direct Nrf2 target [27]. Kurrina et al. used Nrf2-KO mice to demonstrate the direct binding of Nrf2 to the promoter region of miR29a [27]. We suggest here that 4-OI induced upregulation of miR29a via Nrf2 to supress collagen expression. Nrf2 activators such as dimethyl fumarate have been demonstrated to be anti-fibrotic in SSc fibroblasts [37], but their clinical utility may be limited; we suggest that 4-OI could be a novel therapeutic in SSc.

## Figures and Tables

**Table 1:** Primer sequences used in this study.

Gene	Forward	Reverse
HO-1 5′-3′	CTGACCCATGACACCAAGGAC	AAAGCCCTACAGCAACTGTCG
NQO1 5′-3′	TGGAAGCTGCAGACCTGGTG	CCCTTGTCATACATGGTGGCATAC
CCN2 5′-3′	TCCCAAAATCTCCAAGCCTA	GTAATGGCAGGCACAGGTCT
18S 5′-3′	GAATGGCTCATTAAATCAGTT ATGG	TATTAGCTCTAGAATTACCACAGTTATCC

## Data Availability

No extra data is cited in paper.
